# Spontaneous Celiac Artery Pseudoaneurysm in a Patient With Viral Myocarditis: Coincidence or Consequence?

**DOI:** 10.7759/cureus.48970

**Published:** 2023-11-17

**Authors:** Ali Khreisat, Judith Bateman, Marko Kozyk, Kateryna Strubchevska

**Affiliations:** 1 Internal Medicine, Beaumont Health, Royal Oak, USA; 2 Rheumatology, Beaumont Health, Royal Oak, USA; 3 Internal Medicine, Beaumont Hospital, Royal Oak, USA; 4 Internal Medicine, Beaumont Hospital, Ryal Oak, USA

**Keywords:** conventional angiography, ct angiogram, endovascular coil embolization, celiac artery pseudoaneurysm, viral myocarditis, pseudo aneurysm

## Abstract

Spontaneous pseudoaneurysm formation in the celiac artery is a very infrequent occurrence in the absence of trauma or descending aortic dissection. If it continues to progress, it can lead to visceral organ infarction or life-threatening hemoperitoneum. Management is conservative in select cases; however, most patients require an endovascular or surgical approach. The definitive etiology of spontaneous celiac artery pseudoaneurysm remains unclear.

We present an intriguing case of a 67-year-old female who presented to the hospital with sudden chest pain preceded by viral prodromal symptoms. She was discharged as a case of viral myocarditis and was re-admitted the same day with acute abdominal pain. Computed tomography with intravenous contrast showed an enlarging eight-millimeter celiac artery pseudoaneurysm managed with endovascular coil embolization. This case report demonstrates spontaneous celiac artery pseudoaneurysm workup and management. We are also investigating whether a unifying diagnosis exists to explain both viral myocarditis and celiac artery pseudoaneurysm or if both conditions are sporadic occurrences.

## Introduction

Celiac trunk aneurysms and pseudoaneurysms are relatively rare, making up less than four percent of all cases of splanchnic artery aneurysms [1]. Etiologies include congenital malformation, infection, atherosclerosis, blunt trauma, gunshot wounds, or descending aortic dissection [2]. Spontaneous or idiopathic celiac artery pseudoaneurysms are under-reported in the medical literature. We report the first case of spontaneous celiac artery pseudoaneurysm occurring after viral myocarditis. 

## Case presentation

**Figure 1:**
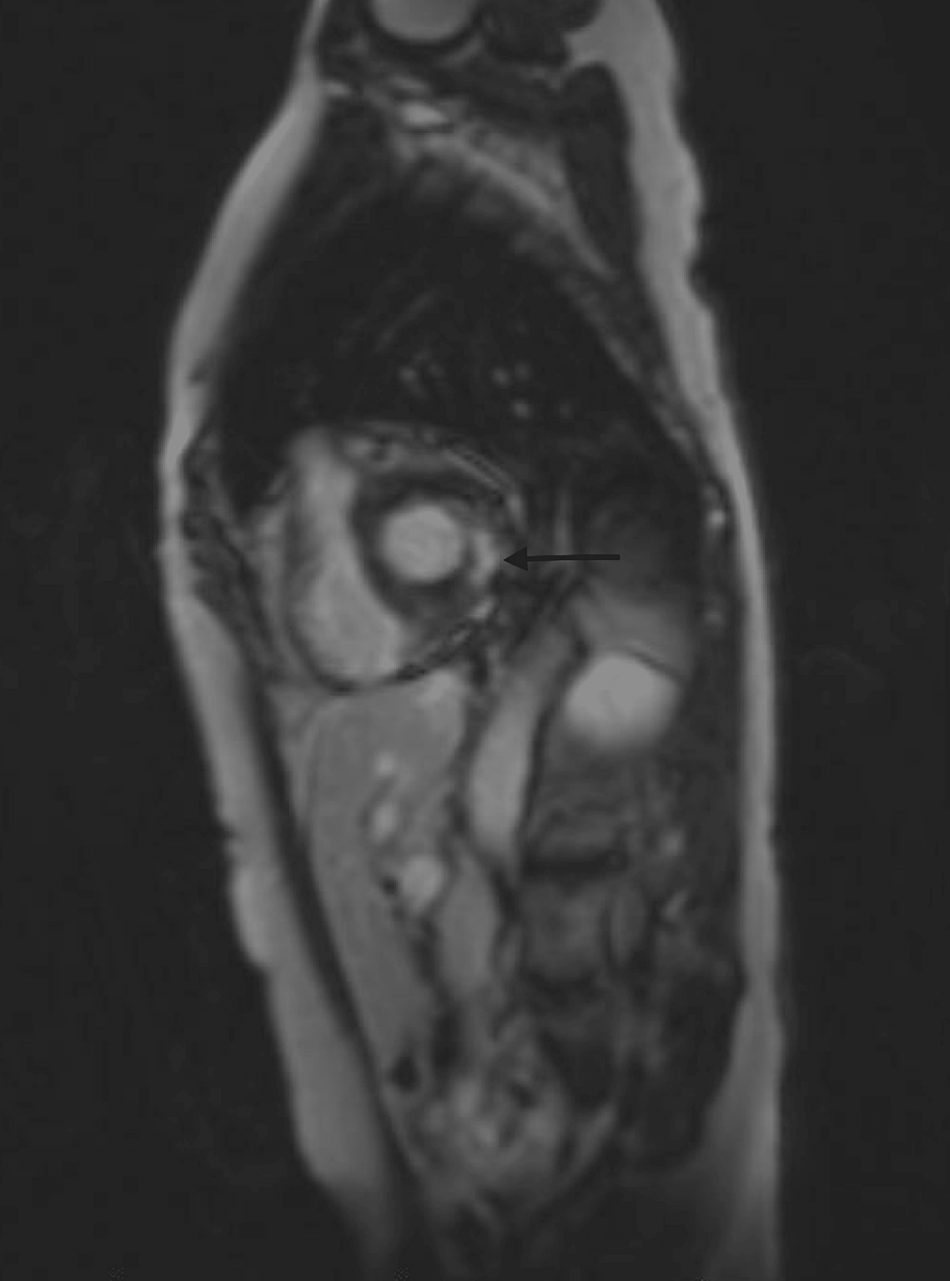
Cardiac magnetic resonance imaging Mid myocardial late gadolinium enhancement in the basal inferolateral wall (arrow) on late gadolinium enhancement images suggestive of myocarditis

A 67-year-old female with a past medical history of achalasia and osteoporosis initially presented to the emergency department with central chest pain that happened at rest, associated with nausea and exertional dyspnea. Two weeks before her chest pain, she had a three-day course of self-limited flu-like symptoms. Upon initial evaluation, she was afebrile and hypertensive at 161/84 mmHg. Her cardiopulmonary physical examination was unremarkable. Laboratory testing showed elevated troponin, trending up with a peak level of 2.39 ng/ml (normal range ≤0.03 ng/mL). Serial electrocardiograms showed normal sinus rhythm without ST segment or T wave changes. Chest/abdomen/pelvis computed tomography with IV contrast did not show signs of aortic dissection, pulmonary embolism, or visceral artery aneurysms. Her chest pain persisted despite intravenous nitroglycerin and heparin. A transthoracic echocardiogram showed a left ventricular ejection fraction of 60%, no valvular vegetation, and no segmental wall motion abnormality. Left heart catheterization through radial access demonstrated minimal coronary artery disease. Cardiac magnetic resonance imaging revealed mid-myocardial late gadolinium enhancement in the inferolateral wall of the myocardium, highly suggestive of myocarditis (Figure 1). The viral panel showed elevated coxsackievirus immunoglobulin M and immunoglobulin G, which is highly suggestive of viral myocarditis. She was discharged from the hospital after three days of presentation. 

**Figure 2:**
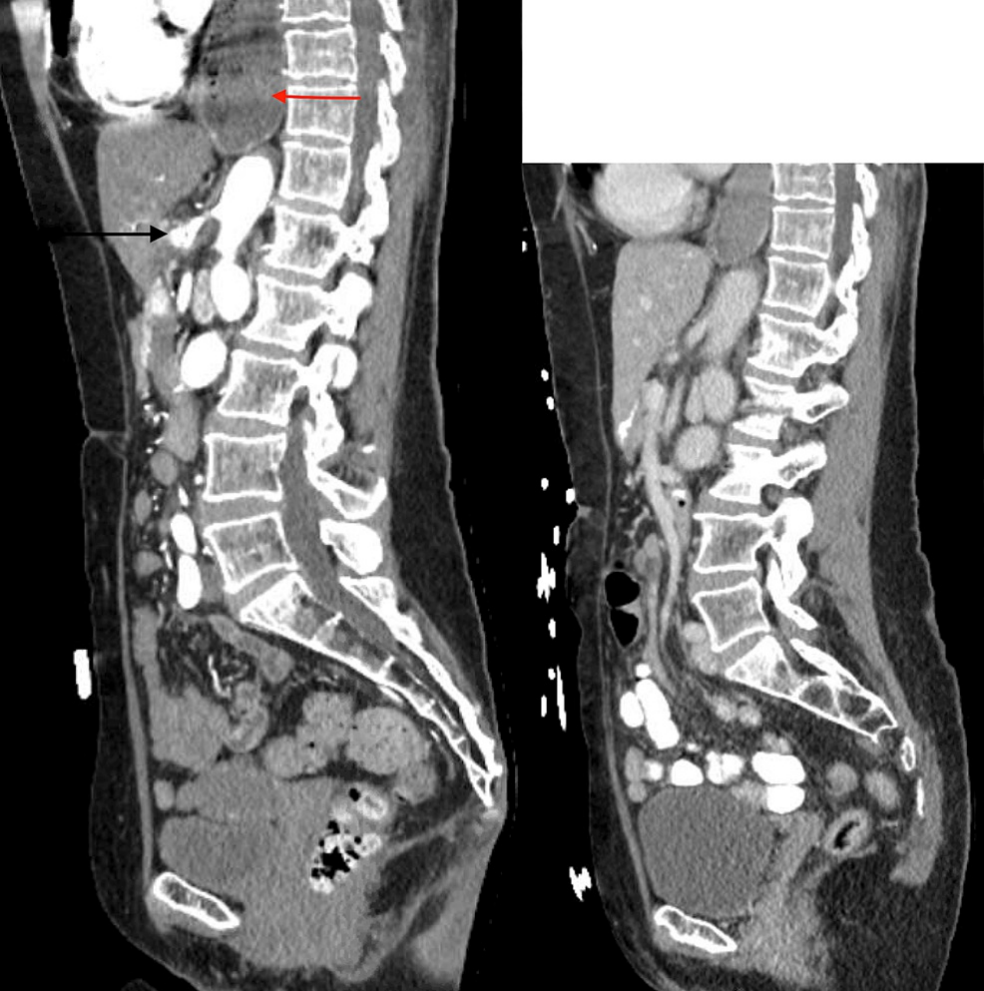
Mid-sagittal computed tomography with intravenous contrast on readmission with abdominal pain (left image) compared to the initial admission computed tomography (right image) The computed tomography scan on the left shows an eight-millimeter celiac artery pseudoaneurysm (black arrow) and a dilated debris-filled esophagus consistent with her known history of achalasia (red arrow). This is new compared to the computed tomography done three days before the onset of her abdominal pain.

**Figure 3:**
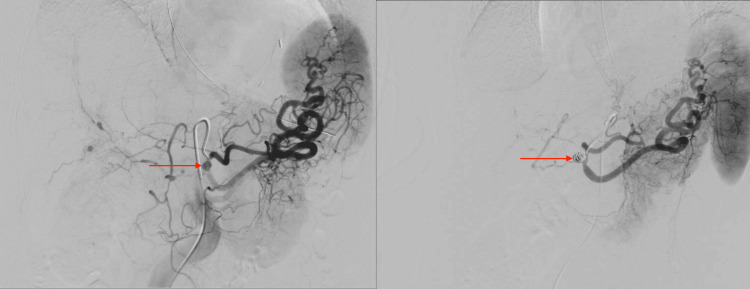
Conventional angiography showing proximal celiac artery pseudoaneurysm (left image) status post coil embolization (right image)

On the day she was discharged, she presented back to the emergency department with sudden epigastric abdominal pain radiating to her back, 9/10 in severity, associated with nausea. She denied a history of abdominal trauma. Vitals signs were stable, and physical examination showed epigastric tenderness on palpation, no guarding, abdominal rigidity, or distension. Computed tomography with IV contrast showed a new celiac artery pseudoaneurysm measuring approximately eight millimeters, which is new compared to the scan performed three days prior (Figure 2). In light of the significant symptomatic nature of her pseudoaneurysm, she underwent a celiac artery angiogram with endovascular coil embolization at the celiac artery bifurcation (Figure 3). Her abdominal pain resolved after the procedure. Laboratory workup showed no leukocytosis, normal C-reactive protein level, negative antinuclear antibody profile, rheumatoid factor, rapid plasma reagin (RPR), and hepatitis B core antibody. There was no bacterial growth on blood cultures. To rule out mycotic aneurysm from septic embolization, a transesophageal echocardiogram was performed, and it revealed no evidence of valvular vegetations. The patient was discharged with a plan for outpatient follow-up with vascular surgery and rheumatology. 

## Discussion

Spontaneous or idiopathic celiac artery pseudoaneurysm formation or dissection is a scarce and under-reported condition in the medical literature, with approximately 34 reported cases between the years 1959 and 2012 [3]. Most cases are associated with significant constant epigastric abdominal pain and physical examination showing epigastric tenderness. We found two case reports in the literature review with a presentation similar to our patient. The first case was managed conservatively [4], and the second was associated with decreased perfusion to the pancreas [5].

It is vital to rule out secondary causes for celiac artery pseudoaneurysms and dissections. Trauma, either blunt or penetrating, is the most common cause of pseudoaneurysm formation [6]. Mycotic (infected) pseudoaneurysms caused by septic embolization from distant sites or involvement of the artery by an adjacent source of infection, usually manifest in the aorta, cerebral vessels, and splanchnic arteries [7], it was ruled out in our patient by negative blood cultures, normal inflammatory markers, and normal transesophageal echocardiogram. Medium vessel vasculitis (like polyarteritis nodosa) causes severe necrotizing vasculitis of multiple visceral arteries, leading to diffuse aneurysmal dilations [8]; our patient's acute presentation and isolated celiac artery involvement makes autoimmune vasculitis unlikely. Viral vasculitis is caused by either direct viral replication leading to inflammation of the vascular wall, as in the case of herpes simplex viruses 1 and 2, or indirectly through cross-reactivity triggering autoimmune vasculitis, as in the case of human immunodeficiency virus (HIV), hepatitis B and C [9]. In our patient, it is unclear if coxsackie B viral myocarditis was directly related to the development of celiac artery vasculitis and pseudoaneurysm formation.

Management of celiac artery pseudoaneurysms depends on the underlying cause. In spontaneous cases, there is a lack of clear guidelines regarding conservative versus endovascular or surgical management. Endovascular management with coil embolization is less invasive for symptomatic patients than open surgical revascularization, which is reserved for patients with life-threatening complications like hemoperitoneum and visceral organ ischemia [10,11].

## Conclusions

Causes for spontaneous celiac artery pseudoaneurysm are yet to be evaluated. While our case report is the first to report it after viral myocarditis, it is unclear if it is a coincidence or a consequence of viral vasculitis. Further studies are needed to investigate the etiologies of spontaneous visceral artery pseudoaneurysms. Our case also highlights the importance of recognizing a potentially life-threatening entity, especially if complicated by pseudoaneurysm rupture or end-organ damage. A multidisciplinary team approach should be implemented for workup, management, and long-term follow-up for patients with spontaneous celiac artery pseudoaneurysm. 
